# The genetic risk of mental health disorders in children from diverse population-based cohorts is modulated by poverty

**DOI:** 10.21203/rs.3.rs-8592737/v1

**Published:** 2026-02-09

**Authors:** Marina Carpena, Enya Nordon, Thais Martins-Silva, Cathy Wyse, Lorna Lopez, Joseph Murray, Luis Rohde, Iná Santos, Alicia Matijasevich, Luciana Tovo-Rodrigues

**Affiliations:** Federal University of Pelotas; Maynooth University; Federal University of Pelotas; Universidade Federal do Rio Grande do Sul

## Abstract

Attention-deficit/hyperactivity disorder (ADHD) and depression share substantial genetic liability, yet the extent to which socioeconomic disadvantage shapes the expression of this genetic risk remains unclear. We investigated whether poverty moderates and mediates associations between polygenic risk scores (PGS) for ADHD and major depressive disorder (MDD) and corresponding symptoms in early adolescence. Data were drawn from two populationbased cohorts: the 2004 Pelotas Birth Cohort (Brazil; N = 3,470) and the Adolescent Brain Cognitive Development (ABCD) Study (United States; N = 10,218). ADHD and depressive symptoms were assessed using caregiver reports (Strengths and Difficulties Questionnaire in Pelotas; Child Behavior Checklist in ABCD). Household income was harmonized and categorized into low (bottom 30%), middle (40%), and high (top 30%) income. PGS were derived using SBayesRC and PLINK2. Linear regression models tested main genetic effects and gene–environment interactions, and mediation analyses quantified indirect effects via poverty, adjusting for age, sex, and ancestry principal components. In Pelotas, ADHD-PGS was associated with ADHD symptoms (β = 0.86, SE = 0.23, p < 0.0001) and emotional symptoms (β = 0.69, SE = 0.24, p = 0.003), with a significant interaction with poverty for ADHD symptoms (pinteraction = 0.024). In ABCD, both ADHD- and MDD-PGS were associated with ADHD and depressive symptoms (all p < 0.001; ΔR^2^ = 0.21–0.48), and gene–environment interactions were observed for all associations (pinteraction < 0.01), indicating attenuated PGS effects under greater socioeconomic disadvantage. Mediation analyses showed that poverty accounted for 7.9–10.2% of the total PGS effect on ADHD symptoms in Pelotas and 4.7–5.3% in ABCD; indirect effects for depressive symptoms were significant only in ABCD (5.3–10.1%). Socioeconomic disadvantage both modifies and partially mediates genetic liability to adolescent psychopathology, suggesting that polygenic risk reflects context-dependent vulnerability shaped by structural conditions.

## INTRODUCTION

Mental health disorders, such as depression and attention-deficit/hyperactivity disorder (ADHD), represent a significant global burden. Depression ranks as one of the leading causes of years lived with disability (YLD) and Disability-Adjusted Life Years (DALYs)^1,2^, while ADHD is among the most prevalent neurodevelopmental conditions in children and adolescents^3^. This is the result of a steady increase in the burden of mental disorders over the last decades^4^, and a trajectory predicted by the WHO to set mental health disorders as the most significant cause of disability worldwide by 2030^5^.

Recent advancements in genome-wide association studies (GWAS) have significantly expanded understanding of the genetic basis of neuropsychiatric conditions such as ADHD and depression^6–9^. These findings have enabled the construction of polygenic scores (PGS), which estimate an individual’s genetic liability for a given trait by combining the effects of associated single-nucleotide polymorphisms (SNPs). PGS has become a powerful tool for assessing genetic contributions to complex psychiatric and behavioral characteristics, offering insights into potential shared genetic pathways. While PGSs capture inherited genetic liability, emerging evidence suggests that gene expression and epigenetic changes—often shaped by environmental exposures—may be moderated by the relationship between adversity and psychopathology^10^. These findings underscore the importance of integrating both inherited and dynamic molecular processes in understanding mental health outcomes. Therefore, despite these advancements, the role of socioeconomic factors, such as poverty, in moderating and/or mediating the effects of genetic risk on these conditions remains underexplored, especially in LMICs.

Poverty is a well-documented environmental stressor that exacerbates the risk for mental health disorders by influencing access to healthcare, environmental exposures, and overall life stressors^11,12^. Early-life deprivation and poverty have been consistently associated with adverse mental health outcomes, including increased risks of anxiety, depression, and behavioural issues^13–15^. In low-and middle-income countries (LMIC), these inequalities are higher.

Approximately 30% of the Brazilian population lives below the poverty line^16^, creating a unique context to study how socioeconomic disadvantage interacts with polygenic risk, leading to mental health outcomes. Schafer et al. (2023)^17^ reported that deprivation longitudinally predicts higher levels of psychopathology in 2,511 children and adolescents participating in a Brazilian high-risk cohort for mental health conditions^18^ Brazil is a country marked by profound socioeconomic inequalities; understanding the interplay between poverty, genetics, and mental health is particularly critical. On the other hand, the United States of America (USA) presents a contrasting scenario, with a lower percentage of the population living below the poverty line (approximately 12% as of 2020, according to the U.S. Census Bureau), yet still grappling with significant disparities in access to mental health care and resources. However, even in a more favourable scenario, the literature has noted that polygenic risk is spatially clustered and amplified by local socioeconomic disadvantage, particularly in areas with low income and high housing vacancy rates^19^. Therefore, comparative studies between Brazil and the USA could provide valuable insights into how genetic and environmental factors interact across diverse socioeconomic and cultural contexts, ultimately informing more targeted and effective interventions to address mental health inequities globally.

We aimed to test whether poverty might moderate and/or mediate the effect of mental health PGS on the susceptibility to ADHD and depressive-related symptoms across different contexts^6,7,9^. This study leverages data from two population-based cohorts to investigate genetic and phenotypic associations while explicitly testing both moderation and mediation, recognizing that poverty may function either as a contextual factor that shapes the expression of genetic liability (G×E moderation) or as a pathway through which genetic influences contribute to socioeconomic disadvantage and, subsequently, to mental health (indirect effect).

## MATERIALS AND METHODS

### Data sources

We used data from the 2004 Pelotas (Brazil) Birth Cohort and the Adolescent Brain Cognitive Development (ABCD) studies.

The 2004 Pelotas Birth Cohort is a population-based longitudinal study that began in 2004. The cohort initially recruited 4,231 newborns born to mothers living in the urban area of Pelotas, a city located in southern Brazil. The participants were followed through multiple stages of childhood and adolescence, with follow-up assessments conducted at various points: at birth (perinatal period, with a 99.2% retention rate), 3 months (95.7%), 12 months (94.3%), 24 months (93.5%), 48 months (92.0%), 6 years (90.2%), 11 years (86.6%), and at 18 years of age (85.0%). The study was designed to assess a range of social, economic, and health factors in both mothers and children. Data from the perinatal period and follow-ups at 6 and 11 years were used in the current analyses, which are described below. The cohort’s methodology has been thoroughly outlined elsewhere^20–22^.

At the 6-year follow-up in 2011, saliva samples were collected from 3,722 participants in the cohort using the Oragene Genotek 250 kit. The DNA was extracted from these samples following the manufacturer’s instructions. Genotyping was performed using the Infinium Global Screening Array v.2 (Illumina). For genetic variants that were not directly genotyped, imputation was performed with SHAPEIT2 and MINIMAC3, using phase 3 data from the 1000 Genomes Project as the reference panel. Rigorous quality control (QC) procedures were applied to ensure data validity. Only SNPs meeting the following criteria were included: (i) fewer than 2% missing genotypes, (ii) an imputation quality score (R^2^) greater than 0.3, and (iii) a minor allele frequency of at least 0.01. These QC steps were executed using PLINK 1.9^23,24^. Furthermore, variants significantly deviating from the Hardy-Weinberg Equilibrium, with a p-value lower than 1e − 6, were excluded. After applying these stringent quality filters, 11,811,746 genetic markers were available for analysis from 3,472 participants. ADHD and depressive symptoms were evaluated through maternal or caregiver reports using the Strengths and Difficulties Questionnaire (SDQ) at age 11. The SDQ has been validated for use in Brazil^25^. It consists of five subscales, each comprising 5 items: conduct problems, emotional problems, hyperactivity/inattention, peer relationship problems, and prosocial behaviour. Items are scored on a 3-point Likert-type scale (*not true, somewhat true*, and *certainly true*), ranging from 0 to 10^26,27^. We used the hyperactivity/inattention and emotional (for depressive symptoms) subscales for the current study. Each subscale ranged from 0 to 10, and we transformed it to a t-score for comparison with the ABCD data.

We used multivariate imputation by chained equations with 1000 imputed datasets to address missing data and to attribute numerical values to the mental health measures. Covariates included: sex, preterm birth, low birth weight, maternal smoking during pregnancy, maternal depression, maternal parity, maternal ethnicity, maternal age, maternal education, and family income. All imputation models were examined for convergence, and detailed information is reported before^28^.

ABCD study (data release 5.0; NDAR-DOI: 10.15154/1503209) is an ongoing, 10-year longitudinal study involving 11,875 individuals, aged 9–10 years at the beginning of the study, followed through to ages 19–20. The study is conducted across 21 sites in the United States and consists of a nationally representative sample. A detailed description of the design and recruitment approach of the ABCD Study is available elsewhere^29^. Briefly, youth participants and their parents were recruited from elementary schools within the catchment areas of these 21 ABCD Study research sites, which encompassed over 20% of the United States’ youth population aged 9 or 10. Multistage probability sampling, based on biological sex, race and ethnicity, socioeconomic status, and urbanicity, was used to yield a sample that closely approximated national sociodemographics, thereby maximising the generalizability of inferences drawn from the sample to the population. We utilized data on genetic and mental health from the second assessment (data release 5.0), which was conducted when participants were 11–12 years old. Child Behavior Checklist (CBCL), a parent-reported measure designed to evaluate various behaviors in children, including anxiety, inattention, aggression, and both externalizing and internalizing behaviors, was used to assess ADHD and depressive symptoms^30,31^. The CBCL consists of 118 items that caregivers complete, rating the degree to which each behavior is proper for their child over the past six months on a 3-point scale (0 = not true, 1 = somewhat true, 2 = very true). The scale is normed by sex, age, informant, and ethnicity and produces several empirically derived syndrome scales^31^. For this study, the focus was specifically on the two DSM-oriented subscales: ADHD problems and depressive symptoms, as we have GWAS data available for this symptomatology. We used their scores on *t* scale to include in our analysis.

Saliva samples were collected at the baseline visit, and DNA was extracted following standard protocols. Genotyping was conducted using the Smokescreen array, designed for genetic studies of addiction and mental health. QC procedures excluded genetic variants with a call rate below 99% and samples with more than 20% missing data or inconsistent identifiers. After QC, genotype data were imputed to the 1000 Genomes Phase 3 reference panel using the Michigan Imputation Server. Genotype probabilities from the imputed data were extracted using QCTOOL v2. (https://www.well.ox.ac.uk/~gav/qctool_v2/) Genetic ancestry was inferred by projecting ABCD participants onto the principal components of the 1000 Genomes Project reference populations, using PLINK v1.90b6.8 with the --pca-clusters flag.

#### Covariables

In both cohorts, covariates were collected during the baseline assessment based on parenting reports and included biological sex, age, skin color, and family income^20,21,32^. Sex designated at birth was classified as male or female, and skin color was self-reported and then categorized as white or mixed (including black, brown, and others). In the 2004 Pelotas Cohort family income in the month before the delivery was collected in Brazilian Real (continuous) and categorized into categories to compare to ABCD data, where almost 30% of the total sample present the lowest family income (what represents the minimun salary of that year), 40% present the most frequent wage, and 30% present the highest family income. In ABCD, income were cathegorizes as: 1. poorest ones received up to the minimum salary annually, what was represented by receiving < 50k/year (in USD); the most frequent salary were cathegorized as receiving ≥ 50k & <100k (in USD), and the highest family income was represented by those receiveng > 100k/year (in USD). In this study, poverty was defined as receiving up to the minimum salary in each context to allow us to compare data. We also included the first 10 principal components of ancestry to control for population stratification in our analysis.

#### Summary statistics for PGS construction

Summary statistics from the largest and most recent GWAS of ADHD^9^ and MDD^7^ were used as discovery data for PGS analysis. ADHD summary statistics included 38,691 cases and 186,843 controls from European, Danish, and Icelandic populations^6^. MDD summary statistics included 688,808 cases and 4,364,225 controls from 29 countries across diverse and admixed ancestries^33^ (Supplementary Table 1 with detailed information).

### Statistical analysis

We described both cohort samples in terms of sociodemographic information. We evaluated potential preexisting inequality in both cohorts using the outcomes of ADHD and MDD phenotypes. Absolute and relative inequalities were calculated by estimating the slope index of inequalities (SII) and the concentration index (CIX), respectively. The SII was calculated using logistic regression and depicts the absolute difference (expressed in percentage points) in the prevalence of the outcome between the top and bottom of the distribution of the socioeconomic variable considered as an inequality dimension. For instance, considering family income, the SII represents the absolute difference in percentage points between the richest and poorest tertiles. In turn, the CIX is an indicator that depicts the relative difference in the prevalence of the outcome according to socioeconomic characteristics. CIX is related to the Gini coefficient, showing how much of the outcome is concentrated in the wealthiest group compared to the poorest group. The CIX measures the concentration of the outcome in each group. The concentration or proportion of people with the outcome should be proportional to the number of people in each group if the outcome is distributed equally. So, for example, if psychiatric symptoms were distributed equally among the richest and the poorest, the 20% poorest and the 20% richest should each account for 20% of all reported cases. Zero values for both SII and CIX represent the absence of inequality; positive values indicate a higher prevalence of the outcome in the better-off group, and negative values indicate a higher prevalence in the worst-off group^34^. Equiplot graphs were used to better illustrate the differences between the subgroups of the inequality dimensions.

PGS were calculated using SBayesRC and PLINK2. SNP effect sizes were estimated using SBayesRC. SBayesRC models SNP effects with a mixture of normal distributions informed by SNP annotations to allow effect size distributions to vary across annotation groups, which demonstrated good fit and prediction in analyzing admixture samples^35^. This tool requires GWAS summary statistics in Conditional and Joint Analysis (COJO) format, LD reference data, and functional annotation data. The LD reference data and functional annotation data were downloaded from github.com/zhilizheng/SBayesRC.The GWAS summary statistics were converted to COJO format before the tidy function of SBayesRC was completed. This function filters SNPs based on SNP sample sizes with fewer than three deviations from the mean, where discrepancies in alleles arise between GWAS summary statistics and LD data. The GWAS summary data were imputed based on LD using the SBayesRC Impute function. Subsequently, the main SBayesRC model was implemented to estimate SNP effect sizes. The output of this model was then used to calculate the PGSs in both the 2004 Pelotas Birth Cohort and the ABCD study (https://abcdstudy.org/) using the --score command in PLINK2.

We used linear regression models to examine the main effects of PGS and their interactions with poverty on the outcomes of interest, while adjusting for the first ten principal components of genetic ancestry (PCA), biological sex, and chronological age. To evaluate the incremental predictive value of the PGS, we calculated the delta R-squared (ΔR^2^) by comparing models with and without the PRS, which reflects the additional variance in the outcome explained by the PGS beyond that accounted for by covariates alone. Additionally, once the genetic overlap between these mental symptoms and socioeconomic disadvantage had been reported before^36,37^., Pathway analysis was employed to calculate natural direct (NDE) and indirect (NIE, mediated) effects, considering poverty as a mediator, while adjusting for uncontrolled confounders. Also, Poisson regression with robust adjustment was used to evaluate the impact of ADHD-, and MDD-PGS on poverty^38^. All inequality assessments, regression, and pathway models were performed using Stata software version 16.1 (StataCorp, College Station, TX, USA), with a significance level of 5%.

#### Ethics statement

The research protocol for all stages of the 2004 Pelotas Birth Cohort Study was approved by the Research Ethics Committee of the Faculty of Medicine at the Federal University of Pelotas and by the Ethics Committee for the Analysis of Research Projects of the University of São Paulo under the numbers 40,602,124 and 889,753. The Brazilian National Commission for Research Ethics also approved the use of genomic data for multifactorial characteristics. Written informed consent was obtained from the mothers or legal guardians. Adolescents also signed an informed consent form at the 11-year follow-up. Cases of severe mental health problems, as identified by the psychologists, were evaluated and, when necessary, were referred to psychiatric or psychological care facilities.

Informed consent and assent were appropriately obtained from the participants in the ABCD study. A data use agreement was established with the National Institute of Mental Health (DAR# 18602), and the rights of participants were protected under the local Institutional Review Boards (IRBs). The authors assert that all procedures contributing to this work comply with the ethical standards of the relevant national and institutional committees on human experimentation and with the Helsinki Declaration of 1975, as revised in 2008.

## RESULTS

### Sample description

Table 1 summarizes the characteristics of the analytical sample, which comprised 3,472 participants from the 2004 Pelotas Birth Cohort. The sample was predominantly composed of males (51.3%), with a mean age of 10.99 years (SD = 0.24). The majority of childcaregivers identified their children as white (67.7%), and 28.7% were classified within the lowest socioeconomic stratum. The average t-scores for symptoms of hyperactivity/inattention and emotional problems were 49.86 (SD = 10.42) and 50.04 (SD = 2.26), respectively (Table 1). The monthly mean family income in the overall sample was R$ 1,451.69 (SD = 33.10). Among participants classified as belonging to the lowest income group, the mean income was substantially lower, at R$ 146.44 (SD = 119.71).

In the ABCD cohort subsample included in the analyses (N = 10,221), 48.8% of participants were female. The mean age was approximately 10 years (SD = 0.64). The majority (54.8%) self-identified as White, and 25.8% were classified as being in the lowest socioeconomic stratum (receiving less than USD 50k per year). Mean symptom t-scores were 53.12 (SD = 5.33) for ADHD and 53.02 (SD = 5.22) for MDD (Table 1).

### Income inequality evidence

**Figure 1:**
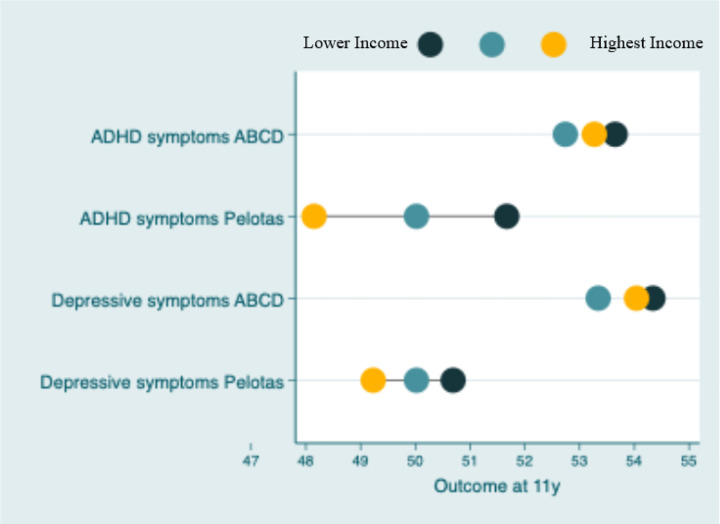
Inequalities in the occurrence of outcomes in both the 2004 Pelotas Birth Cohort and the ABCDStudy

An income gradient was observed for both ADHD/hyperactivity/inattention symptoms and depressive symptoms/emotional problems at age 11 across the two cohorts, with higher symptom scores among children from lower-income households for both traits and both cohorts (Figure 1, detailed information available in Supplementary Table 2). In the 2004 Pelotas Birth Cohort, participants in the lowest income group exhibited the highest mean ADHD scores (M = 51.67, SD = 10.10). There was a significant trend where the highest household income co-occurs with the lowest ADHD symptomatology. Those in the highest income group presented the lowest ADHD scores (M = 48.15, SD = 9.26). The absolute (SII) and relative (CXI) inequality coefficients were −5.14 (95% CI: −6.43 to −3.84) and −1.5 (95% CI: −1.9 to −1.1), respectively, indicating a substantial pro-poor inequality (i.e., refers to situations where worse outcomes are concentrated among the poorest group). In the ABCD cohort, this socioeconomic inequality was present but less pronounced, with an SII of −1.42 (95% CI: −1.80 to −1.03) and a CIX of −0.4 (95% CI: −0.5 to −0.3). Subjects from the highest income stratum had the lowest mean ADHD symptom scores (M = 53.65, SD = 5.76); however, the difference was not substantial, and there was no trend in symptom scores with increasing household income.

In the Pelotas cohort, children from lower-income families reported higher levels of emotional symptoms, with a trend toward decreasing scores to a mean of 49.23 (SD = 9.80) in the highest-income group. The SII and CIX corroborated evidence of socioeconomic inequalities with an SII of −2.28 (95% CI: −3.59 to −0.97) and a CIX of −0.7 (95% CI: −1.1 to −0.3), indicating that emotional problems were concentrated among children from lower-income families. In the ABCD cohort, no trend was observed; however, children from higher-income households reported lower depressive symptom scores (M = 54.34, SD = 6.71). Specifically, within the ABCD sample, children from families earning less than USD 50,000 annually presented higher mean scores for depressive symptoms. In contrast, those from households with annual incomes exceeding USD 100,000 had the lowest mean scores (depression: M = 53.34, SD = 5.39). The inequalities metric showed a smaller magnitude, with an SII of −1.61 (95% CI: −2.04 to −1.18) and a CIX of −0.4 (95% CI: −0.5 to 0.3), also reflecting a pro-poor inequality pattern.

### PGS and phenotype associations

Analyses from the 2004 Pelotas Birth Cohort and the ABCD Study also revealed robust associations between ADHD-PGS and MDD-PGS with mental health outcomes. ADHD-PGS was positively associated with ADHD symptoms in both Pelotas (β = 0.23, SE = 0.06, p < 0.001, ΔR^2^ = 0.38%) and ABCD (β = 0.41, SE = 0.07, p < 0.001, ΔR^2^ = 0.38). ADHD-PGS also showed significant associations with emotional symptoms (Pelotas: β = 0.15, SE = 0.05, p = 0.003, ΔR^2^ = 0.26%; ABCD: β = 0.34, SE = 0.07, p < 0.001, ΔR^2^ = 0.21%). MDD-PGS was significantly associated with emotional symptoms in both cohorts, with a larger effect in ABCD (β = 0.51, SE = 0.07, p < 0.001, ΔR^2^ = 0.48%) than in Pelotas (β = 0.15, SE = 0.05, p = 0.004, ΔR^2^ = 0.46%). Associations between MDD-PGS and ADHD symptoms reached significance only in ABCD (β = 0.39, SE = 0.06, p < 0.001, ΔR^2^ = 0.36%), while the association in Pelotas was marginal (β = 0.11, SE = 0.06, p = 0.082, ΔR^2^ = 0.12%) (Table 2).

### PGS and its association with poverty

There is evidence of the association between higher scores in ADHD- and MDD-PGS and increased likelihood of poverty (Table 2). In the Pelotas cohort, higher ADHD-PGS was associated with greater poverty exposure (PR = 1.08, p = 0.003, ΔR^2^ = 0.12%), as was MDD-PGS (PR = 1.09, p = 0.003, ΔR^2^ = 0.13%). In the ABCD cohort, similar patterns were observed: ADHD-PGS co-occurred with poverty (PR = 1.11, p < 0.001, ΔR^2^ = 0.17%) and MDD-PGS was also significantly associated with poverty (PR = 1.10, p < 0.001, ΔR^2^ = 0.15%).

### PGS and poverty interaction

**Figure 2:**
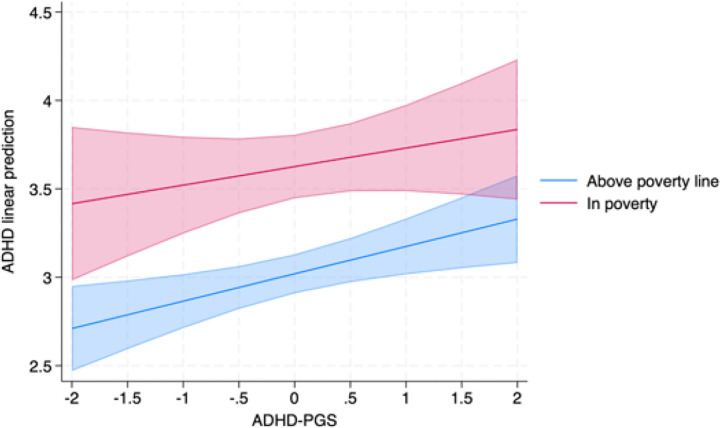
Moderation effect of poverty on the association between ADHD-PGS and ADHD symptoms using 2004 Pelotas Birth Cohort (N=3470, Brazil).

We further tested whether poverty could modify the genetic susceptibility to hyperactivity/inattention symptoms and depressive/emotional problems symptoms. In Pelotas, ADHD-PGS showed a significant interaction with poverty for ADHD symptoms (p-interaction = 0.024, ΔR^2^ = 0.38%), but not for emotional symptoms (p-interaction = 0.153). A one-standard-deviation increase in the ADHD-PGS was associated with a more pronounced increase in ADHD symptom levels among adolescents living in poverty (β = 0.77, 95% CI: 0.06; 1.49), compared to their peers not exposed to poverty (β = 0.48, 95% CI: 0.08; 0.87). Also, we can see perfectly how low income might be correlated with higher symptomatology, even between those with lower ADHD-PGS (Figure 2).

**Figure 3:**
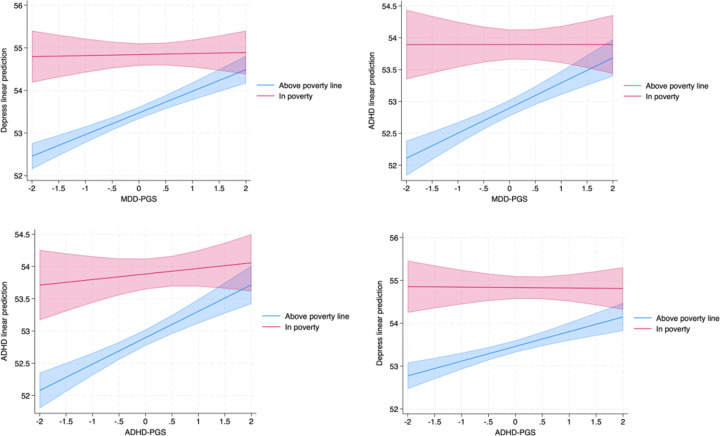
Moderation effect of poverty on the association between a) major depressive disorder polygenic score (MDD-PGS) and depressive symptoms, b) MDD-PGS and attention deficit hyperactivity (ADHD) symptoms, c)ADHD-PGS and ADHD, and d) ADHD-PGS and depressive symptoms, using Adolescent Brain Cognitive Development Study (ABCDStudy, N=10218, USA).

In the ABCD cohort, the interaction terms indicated stronger genetic effects among adolescents exposed to poverty compared to their counterparts, and the same prediction pattern. For example, the ADHD-PGS × poverty interaction was significant for ADHD symptoms (β-interaction = −0.32, p = 0.009, ΔR^2^ = 0.38%) and depressive symptoms (β-interaction = −0.35, p = 0.011, ΔR^2^ = 0.21%). MDD-PGS also interacted significantly with poverty to predict both ADHD (β-interaction = −0.39, p = 0.002, ΔR^2^ = 0.36%) and depressive symptoms (β-interaction = −0.48, p = 0.001, ΔR^2^ = 0.48%). Figure 3 shows clearly that socioeconomic disadvantage amplifies mental health symptoms; however, the expression of genetic risk is stronger among the non-poorest ones in the ABCD cohort.

### PGS and poverty mediation analysis

To test whether poverty might mediate the association between genetic ADHD susceptibility and hyperactivity/inattention symptoms, we performed a mediation analysis presented in Table 3. It revealed that while NDE of ADHD- and MDD-PGS on both ADHD and depressive-related outcomes were substantial in both the Pelotas and ABCD cohorts, there was also evidence of statistically significant NIE through poverty. For PGS-ADHD, the NIE via poverty accounted for 7.9% of the total effect in Pelotas (β =0.02, p = 0.025) and 5.3% in ABCD (β =0.04, p = 0.002), indicating that early-life socioeconomic conditions partially mediated the genetic liability to ADHD.

For PGS-MDD, poverty mediated 10.2% of the effect on ADHD in Pelotas (β =0.03, p=0.021) and 4.7% in ABCD (β =0.03, p=0.004). Regarding depressive-related outcome, the NIE was modest but significant only in ABCD (β =0.05, p=0.001), while it was negligible and non-significant in Pelotas (β=0.01, p=0.263).

## DISCUSSION

This study investigated whether poverty moderates or mediates genetic susceptibility to mental health problems in early adolescence, drawing on data from two large, population-based cohorts in Brazil and the United States. Across both cohorts, lower household income was consistently associated with elevated levels of ADHD and depressive symptoms, underscoring a pronounced socioeconomic gradient in adolescent mental health. PGS for ADHD and MDD showed robust associations with their respective symptom domains, reflecting underlying genetic liability. Gene–environment (G x E) interaction analyses revealed that the influence of socioeconomic disadvantage on the genetic predisposition, especially for ADHD symptoms, was observed across Brazilian and U.S.A samples. The mediation analysis showed that poverty at birth might mediate 4–10% of the total effect of PGS on their outcome. These findings suggest that socioeconomic adversity may exacerbate psychiatric symptoms and shape the expression of genetic vulnerability, underscoring the importance of integrating genetic liability and environmental context in models of adolescent mental health.

Our findings indicated that poverty is consistently associated with greater symptom severity of ADHD and MDD across both the 2004 Pelotas Birth Cohort and the ABCD Study. These results align with prior evidence demonstrating that children from socioeconomically disadvantaged backgrounds are disproportionately affected by mental health difficulties^39,40^. While aligning with prior evidence identifying poverty and inequality as critical impediments to healthy child development^41,42^, our findings advance this literature by demonstrating that the effects of socioeconomic disadvantage are not homogeneous. These findings are in line with gene-environment interaction research showing that genetic liability for ADHD, such as the COMT Val158Met polymorphism, may confer greater vulnerability in contexts of early-life adversity^43^. We observed the same increased risk for the poorer ones; however, we add that the current literature suggests that polygenic scores might serve as a better phenotype-prediction model only in a socioeconomic-advantage context. Similarly, Wickrama et al. (2021)^44^ documented that poverty interacts with genetic vulnerability to increase risk for developmental and health impairments, while also contributing to persistent educational disparities and cumulative disadvantage over the life course. However, previously conducted investigations with the ABCD sample reported apparent main effects of both polygenic risk and poverty, but did not consistently find significant interactions (with p-values ranging from 0.06 to 0.48 in some cases), suggesting a potential confounder bias due to ancestry^45^. Indeed, even in studies where interaction terms were not statistically significant, such as in Mooney et al. (2023)^46^, more substantial environmental effects were observed among individuals with lower genetic risk — a pattern that was similarly evident in both the Pelotas and ABCD cohorts in this study.

Interestingly, our study found a dual role for poverty: both a context that amplifies symptoms and a potential pathway through which genetic risk is expressed, highlighting the complex interplay between biological predisposition and structural determinants. Recent Mendelian randomization analyses, integrating multiple indicators of poverty such as household income, occupational status, and neighbourhood deprivation, further support these associations^47^. Specifically, the authors provide evidence for a bidirectional causal relationship between poverty and ADHD, and a unidirectional causal effect of poverty on MDD^48^. The most consistent indirect and interaction effects were observed for the association between the ADHD PGS and ADHD symptoms, which might indicate consistency with the estimated causal link between poverty and ADHD reported before, where Marchi et al. (2024)^48^ point out that it may be partially confounded by horizontal pleiotropy, warranting cautious interpretation of its magnitude. This pattern likely reflects shared genetic influences on socioeconomic position and mental health outcomes, suggesting that socioeconomic disadvantage is partly captured by GWAS-derived polygenic scores.^48^. Together, this supports the notion that genetic predisposition and structural disadvantage jointly shape developmental mental health risk for psychopathology.

The proportion of the total effect mediated by poverty was modest, particularly for depressive symptoms, and reached statistical significance only in the U.S.A. cohort. Although these findings align with prior evidence suggesting both moderating and mediating roles of socioeconomic adversity in depression (30), they partially diverge from recent Mendelian randomization results indicating a robust causal effect of socioeconomic disadvantage on depression, independent of confounders such as cognitive ability^48^. Importantly, our analyses were conducted in large, sociodemographically diverse samples, enhancing the generalizability of findings related to structural inequalities. These results underscore the potential utility of integrated intervention strategies that address both genetic vulnerability and social determinants such as poverty. Moreover, prior reviews have highlighted how G×E interactions may obscure the detection of genetic associations and contribute to interindividual differences in sensitivity to environmental stressors and response to behavioral interventions (31). Collectively, these findings reinforce that genetic expression is context-dependent.

This study has limitations that should be considered when interpreting the findings. Mental health symptoms were assessed using different instruments across cohorts: the SDQ in Pelotas and the CBCL in ABCD. Although both capture related symptom dimensions and are widely used in population-based research, they reflect somewhat different constructs, which may influence comparability, especially for depressive-like symptoms (emotional difficulties SDQ-subscale vs. depressive CBCL-subscale). Additionally, mental health data in both cohorts relied solely on parent reports, potentially introducing reporter bias and limiting clinical precision. Poverty measures were harmonized to enable cross-cohort comparisons; however, they may not fully capture the local nuances of poverty, particularly in Pelotas, where income variability is greater. Finally, both cohorts had a highly admixed genetic background, which may complicate the transfer of PGS. To mitigate this, we employed a multi-ancestry PGS methodology and utilized the available multi-ancestry MDD-GWAS; however, residual confounding due to complex local ancestry and linkage disequilibrium patterns may remain.

Despite the limitations mentioned, we identify several key strengths of our study that enhance the robustness and relevance of our findings. The use of harmonized data from two large, population-based cohorts in Brazil and the U.S.A. is a strength of our study, as it enabled us to replicate findings across distinct sociocultural contexts and enhance their generalizability. We observed that ADHD- and MDD-PGS were robustly associated with corresponding symptom dimensions, reinforcing the substantial contribution of common genetic variation to the etiology of these psychiatric traits, and a highly corroborating finding supporting evidence of shared genetic architecture between these conditions (e.g., Demontis et al., 2022^49^; Wray et al., 2018^50^).

Therefore, our findings extend this literature by showing that socioeconomic adversity, which is partially captured by GWAS-derived polygenic scores, affects the interpretation and predictive utility of PGS for identifying susceptibility to common psychiatric conditions such as ADHD and MDD. This study contributes to a growing body of evidence indicating that mental health outcomes, including ADHD and MDD, emerge from the dynamic interplay between genetic liability and environmental exposures, particularly socioeconomic disadvantage. Our findings emphasize that genetic influences on psychopathology are not uniformly expressed across contexts but are significantly moderated by environmental conditions such as poverty. His gene–environment interplay highlights the importance of early detection strategies and policies that mitigate socioeconomic disparities, particularly for individuals with heightened genetic vulnerability. Future research should aim to elucidate these mechanisms through longitudinal studies and interventional trials, thereby informing precision-based approaches to mental health prevention and care.

## Supplementary Material

Supplementary Files

This is a list of supplementary files associated with this preprint. Click to download.

- supplementarytables.docx

## Figures and Tables

**Table 1.** Description of Participants Included from the 2004 Pelotas Birth Cohort and the Adolescent Brain Cognitive Development (ABCD) Study.

	Pelotas (N=3472)	ABCD (N=10221)
Biological sex, female N (%)	1,692 (48.73)	2,198 (48.78)
Children age, mean (sd)	10.99 (0.24)	9.91 (0.64)
Skin color, white N (%)	2,349 (67.68)	5,599 (54.78)
Poverty one, N (%)	996 (28.7)	2,641 (25.84)
ADHD, mean (sd)	49.86 (10.42)	53.12 (5.33)
MDD/emotional, mean (sd)	50.04 (10.49)	53.78 (5.93)

Note: Attention deficit hyperactivity disorder (ADHD) and major depressive disorder/emotional (MDD) symptoms

**Table 2.** Polygenic score (PGS) effects and its interaction term leading outcomes, attention deficit hyperactivity disorder (ADHD) and major depressive disorder/emotional symptoms (MDD) for both the 2004 Pelotas Birth Cohort and Adolescent Brain Cognitive Development Study (ABCD) samples.

		The 2004 Pelotas Birth Cohort						ABCDStudy					
Exposure	Outcome	N	badjusted* (SE)	p-value	interaction term	p-interaction	ΔR2 (%)	N	badjusted* (SE)	p-value	interaction term	p-interaction	ΔR2 (%)
ADHD-PGS	**ADHD symptoms**	3470	0.23 (0.06)	**<0.0001**	−0.0001	**0.023**	**0.38**	10218	0.41 (0.07)	**<0.0001**	−0.32	**0.009**	**0.38**
	**MDD/emotional symptoms**	3470	0.15 (0.05)	**0.003**	−0.0001	0.153	**0.26**	10217	0.34 (0.07)	**<0.0001**	−0.35	**0.011**	**0.21**
	**Poverty** *	3471	1.08 (0.03)	**0.003**	N/A	N/A	**0.12**	10217	1.11 (0.02)	**<0.0001**	N/A	N/A	**0.17**
													
MDD-PGS	**ADHD symptoms**	3470	0.11 (0.06)	0.082	−3.48e-06	0.940	0.12	10218	0.39 (0.06)	**<0.001**	−0.39	**0.002**	**0.36**
	**MDD/emotional symptoms**	3470	0.15 (0.05)	**0.004**	0.00002	0.583	**0.46**	10217	0.51 (0.07)	**<0.001**	−0.48	**0.001**	**0.48**
	**Poverty** *	3471	1.09 (0.03)	**0.003**	N/A	N/A	**0.13**	10217	1.10 (0.02)	**<0.001**	N/A	**N/A**	**0.15**

Note: adjusted for the first 10 principal components of ancestry, biological sex, and age;

* Prevalence Ratio (PR) were presented instead of beta

**Table 3.** Natural direct (NDE) and indirect effects (NIE) (mediated by poverty) of polygenic scores (PGS) for Attention-Deficit/Hyperactivity Disorder (ADHD) and Major depressive disorder (MDD) on ADHD and emotional/depressive symptom outcomes in the 2004 Pelotas Birth Cohort and the Adolescent Brain Cognitive Development (ABCDStudy)

			Pelotas				ABCDStudy			
			badjusted*	(SE)	%	P	badjusted*	(SE)	%	P
PGS-ADHD	ADHD	NDE	0.28	0.10	92.06	**0.003**	0.70	0.12	94.69	**<0.0001**
		NIE	0.02	0.01	7.94	**0.025**	0.04	0.01	5.31	**0.002**
	MDD	NDE	0.20	0.08	96.01	**0.010**	0.56	0.13	89.89	**<0.0001**
		NIE	0.01	0.01	3.99	0.167	0.06	0.02	10.11	**<0.0001**
PGS-MDD	ADHD	NDE	0.24	0.10	89.81	**0.021**	0.64	0.12	95.28	**<0.001**
		NIE	0.03	0.01	10.19	**0.021**	0.03	0.01	4.72	**0.004**
	MDD	NDE	0.33	0.08	98.01	**<0.001**	0.83	0.13	94.72	**<0.001**
		NIE	0.01	0.01	1.99	0.263	0.05	0.01	5.28	**0.001**

Note: adjusted for the first 10 principal components of ancestry, biological sex, and age;

## Data Availability

Applications to use the Pelotas data can be made by contacting the research team of the 2004 Pelotas Birth Cohort Study (see: https://www.epidemioufpel.org.br/site/content/faculty/) and submitting the application form available at https://www.epidemio-ufpel.org.br/site/content/studies/formularios.php. A list of administered questionnaires at each wave can be found here: https://www.epidemio-ufpel.org.br/site/content/coorte_2004-en/questionnaires.php. Approved requests will receive a dataset with anonymized participant identifiers and requested variables. ABCD data have been deposited in The National Institute of Mental Health Data Archive (NDA) (https://doi.org/10.15154/1526524) (54). Data used in the preparation of this article were obtained from the Adolescent Brain Cognitive Development (ABCD) Study (https://abcdstudy.org), held in the NIMH Data Archive (NDA). This is a multisite, longitudinal study designed to recruit more than 10,000 children age 9 to 10 and follow them over 10 y into early adulthood. The ABCD Study^®^ is supported by the National Institutes of Health and additional federal partners under award numbers U01DA041048, U01DA050989, U01DA051016, U01DA041022, U01DA051018, U01DA051037, U01DA050987, U01DA041174, U01DA041106, U01DA041117, U01DA041028, U01DA041134, U01DA050988, U01DA051039, U01DA041156, U01DA041025, U01DA041120, U01DA051038, U01DA041148, U01DA041093, U01DA041089, U24DA041123, U24DA041147. A full list of supporters is available at https://abcdstudy.org/federal-partners.html. A listing of participating sites and a complete listing of the study investigators can be found at https://abcdstudy.org/consortium_members/. ABCD consortium investigators designed and implemented the study and/or provided data, but did not necessarily participate in the analysis or writing of this report. This manuscript reflects the views of the authors and may not reflect the opinions or views of the NIH or ABCD consortium investigators.
